# Effects of Multi-Walled Carbon Nanotube Dosages and Sonication Time on Hydration Heat Evolution in Cementitious Composites

**DOI:** 10.3390/ma16227246

**Published:** 2023-11-20

**Authors:** Barbara Klemczak, Eryk Goldmann, Małgorzata Gołaszewska, Marcin Górski

**Affiliations:** 1 Department of Structural Engineering, Faculty of Civil Engineering, Silesian University of Technology, 44-100 Gliwice, Poland; barbara.klemczak@polsl.pl (B.K.); marcin.gorski@polsl.pl (M.G.); 2 Department of Building Processes and Building Physics, Faculty of Civil Engineering, Silesian University of Technology, 44-100 Gliwice, Poland; malgorzata.golaszewska@polsl.pl

**Keywords:** cementitious composite, multi-walled carbon nanotubes, sonication, hydration heat, isothermal calorimetry

## Abstract

This study aimed to investigate the heat generated during the hydration process in cementitious composites containing multi-walled carbon nanotubes (MWCNTs). The cumulative heat release and heat flow of these cementitious composites were measured over a period of 168 h using isothermal calorimetry. Three different MWCNT dosages, 0.05 wt%, 0.1 wt%, and 0.2 wt%, along with two different sonication times for the solution, which were 20 min and 60 min, were applied in the experimental program. The results reveal that the incorporation of MWCNTs and the use of a naphthalene-based superplasticizer to disperse the nanotubes generally led to a reduction in heat emission during the early stages of hydration, a lower first peak value in the initial stage of hydration, and a significant delay in the acceleration period compared with the reference sample lacking this superplasticizer. Furthermore, the results demonstrate that both the dosage of multi-walled carbon nanotubes (MWCNTs) and the sonication time have an impact on the heat emission and hydration process since the same amount of superplasticizer was applied to all pastes. An increase in the MWCNT dosage led to a decrease in the rate of hydration heat at the main peak for all pastes. Additionally, longer sonication times resulted in lower values of heat generated, reduced main peak values in the heat rate evolution, and generally extended delays in the occurrence of the main peak.

## 1. Introduction

The addition of carbon nanotubes (CNTs) into a cement matrix is a popular direction for research in the area of modern construction materials [1]. The improvement of mechanical strength and thermal and electrical conductivity allows for the exploration of various applications of cement nanocomposites, including smart materials used for strain monitoring, heat sinks, and thermal materials [2,3,4,5,6,7]. Conductive carbon materials, such as carbon nanotubes, can also be introduced into perovskite solar cells, which are the most promising solar cells due to advantages such as high-power conversion efficiency, low cost, and ease of fabrication [8,9]. The mechanical strength improvement of a cement material is linked with the growth of the C-S-H phase, especially at an early age, and the nano-reinforcement effect, while conductivity, both electrical and thermal, is related to the internal pathways that CNTs create inside the cement matrix [10,11]. For the abovementioned properties to be exploited in the most effective way, a proper dispersion of carbon nanofiller is mandatory [12,13].

Carbon nanomaterials are usually added to cement via a water suspension [12,13]. To provide a proper distribution of a carbon nanomaterial in the volume of a cement matrix, this suspension is prepared with the use of various dispersion-aiding techniques. The most popular techniques combine the addition of surfactants, superplasticizers, and SiO2 nanoparticles (nano-silica) and mixing using high-energy ultrasound. Surfactants adhere to CNTs and separate them on a molecular level using steric or electrostatic repulsive mechanisms to counteract Van der Waals forces. A similar mechanism is utilised with nano-silica, whereby the spherical grains separate carbon nanotubes by mechanically pushing them away from each other [11].

Additionally, the application of ultrasound mixing provides more mechanical energy to aid with breaking up the agglomerates. The process of sonication is usually described with either the total time of the process or cumulative energy passed into the suspension [13,14]. The sonication parameters and types of surfactants used vary between researchers mainly because the type and size of carbon nanotubes are decisive factors for the combination of a specific chemical and method’s effectiveness. Differences in the chemical composition of concrete admixtures can also cause them to perform differently with various types of carbon nanotubes.

Despite the abovementioned differences, a combination of a surfactant and sonication is often claimed to give the best results in terms of aiding the dispersion of carbon nanotubes in water [12,13,15,16].

Even though most of the research on carbon nanotube–cement composites has focused on mechanical strength or smart material properties, some attention has also been given to understanding the hydration kinetics of these materials and the effect of carbon nanotubes on the early-age behaviour of the cement matrix.

Since carbon nanotubes (CNTs) can assist in the formation of hydration products, they can also influence the heat generated during the hydration process. Moreover, as they can be dispersed in superplasticizers, some of the superplasticizers might not be fully used for dispersion, therefore influencing the hydration kinetics [17]. Furthermore, the effect of CNTs on hydration has been attributed to a physical rather than chemical phenomenon. The authors of [17] concluded that CNTs provide nucleation points for hydration products to grow on even if a superplasticizer is also incorporated into the mix. The nucleation effect was predominant in increasing the main peak of C_3_S hydration compared with the plain cement paste. Finally, it was also concluded that the surface area of CNTs, which is available to hydration products, is the key factor for the nucleation effect.

Furthermore, the internal network of dispersed nanotubes can not only enhance electrical and thermal conductivity but also act as scaffolding for growing hydration products and bridging effects between hydrates [18,19]. Additionally, CNTs can act as nucleation points for cement hydrates, therefore promoting the growth of the C-S-H phase and improving the hydration process. Hence, in [16], it was observed that single-walled carbon nanotubes dispersed on cement grains restricted the formation of C_3_A hydration products in the early stage of the hydration reaction by occupying the nucleation sites on the cement grains, therefore physically blocking the growth of C_3_A products. This also led to more C_3_S being exposed and, in consequence, a larger initial formation of the C-S-H phase and higher peak 2 in the calorimetric results. The hydration products also formed close to the carbon nanotubes, which were used as nucleation points. Using SEM imaging of different stages of the early hydration reaction, a supportive effect of CNTs on the growing C-S-H products was noticed.

Tafesse and Kim [20] performed a series of experiments to find the influence of CNTs dispersed with nano-silica fume on the hydration process. They concluded that the influence is not significant in chemical terms and neither activates nor hinders hydration. The change in heat evolved was at a level of approximately 3%, and process delays of 6 h and 10 h were concluded to occur because of superplasticizer addition. The only influence on the hydration process was of a physical nature when CNT and silica fume agglomerations trapped water in their structures, therefore increasing autogenous shrinkage. Next, Tafesse and Kim [18] claimed that pure CNTs can only adsorb superplasticizers used for their dispersion in an insignificant manner and form no functional groups with these substances. They act solely as solid nanofillers with no chemical interactions with cement components. The authors also concluded that CNTs have little effect on hydration from a chemical point of view. They can, however, hinder the hydration reaction in a physical way by trapping water in between agglomerations, leaving less water to participate in the reaction.

The experiment performed by Park and Choi [21] shows that the setting time of a cement composite with CNTs reduced proportionally to the amount of carbon nanotubes added by 2.4% and 3.0% for initial and final setting times, respectively. Therefore, it was concluded that CNTs accelerate the hydration process in the early stages by reducing its induction period; however, the influence in later hydration stages was not significant between different CNT amounts.

MacLeod et al. [15] compared the effect of carbon nanotubes with and without superplasticizers and various dispersion qualities on hydration kinetics. Their results indicate that the CNT dosage, not dispersion quality, has the dominant role in terms of the CNTs’ influence on hydration kinetics. For samples with pure CNTs, the hydration process was accelerated at early ages, with a 16.6% growth in the peak heat value and increased heat generation during the 24 and 72 h time periods. The addition of a superplasticizer hindered this effect, mainly in the first 24 h.

Next, Leonavicius et al. [22] assessed the influence of CNTs dispersed in carboxyl-methyl cellulose on the setting time and hydration heat of cement composites. They linked it with the reduction in pH caused by the nanofiller, which hindered the hydration process, and prolonged the setting time by approximately 100% and the induction period by up to 10 h. Karpova et al. [23] used a similar composition of carbon nanotubes dispersed in carboxyl-methyl cellulose with a polycarboxylate ether superplasticizer. Their results show that the application of that complex admixture prolonged hydration in each phase and led to a decrease in heat flow by 35% in the acceleration period and total heat emission by 26% after 48 h. Stynoski et al. [24] observed a relatively small effect of functionalised carbon nanotubes on the hydration process, with differences in heat generation of approximately 6%. Jung et al. [17] noticed an insignificant influence of CNTs on hydration heat, with a lower than 10% difference in cumulative heat; however, they pointed out the bridging effect of CNTs between hydration products, which was apparent in SEM images.

An investigation performed by Li et al. [25] on the influence of dosages of CNTs at levels of 0.1 wt%, 0.3 wt%, and 0.5 wt% showed that carbon nanotubes accelerate the hydration rate, shifting the heat peaks to 3.06%, 8.69%, and 4.65% earlier compared with control samples, respectively. Moreover, the sample with 0.1 wt% of CNTs significantly reduced the total cumulative heat, while at 0.5 wt%, the total cumulative heat was nearly the same as for the control sample. The CNT dosage of 0.3 wt% was deemed the best as it exhibited a balanced result in terms of the nucleation effect, water absorption factor, and aggregation factor among the tested dosages.

The delayed hydration of multi-walled carbon nanotube (MWCNT) composites has also been documented in studies [26,27,28]. Additionally, research in [29] revealed that carbon nanotubes can enhance the dissolution rates of adjacent C_3_S and C_2_S by up to 6%. However, it was simultaneously observed that the impacts of CNTs and superplasticizers can be distinguished and quantified. Superplasticizers used in CNT-enhanced composites were found to delay the initial hydration of CNT-reinforced cement paste due to the steric hindrance effect influenced by the content of both CNTs and superplasticizers. The aforementioned study [29] also mentioned that despite CNTs being known as non-chemically reactive in the hydration process, their effects can be identified and quantified. The influence of CNTs on the hydration process is linked to the growth of calcium silicate hydrates (CSHs) on the surfaces of the CNTs, and the resulting bridging effect is closely associated with the microstructures of the cement [30]. Furthermore, the same study [29] proposed a cement hydration model based on multiphase voxels and a novel dissolution rate to simulate the impact of CNTs on the hydration of cement pastes.

The mentioned literature reports, even if their results vary in assessing the overall effect of carbon nanotubes on the hydration process, predominantly conclude that the effect—either the acceleration or deceleration of hydration—is not very significant. Furthermore, it is likely that for non-functionalised carbon nanotubes, their influence on hydration mechanics is more of a physical phenomenon than a chemical one. However, the available results have most often focused on one factor related to the dispersion method or the amount of carbon nanotubes added. The presented study focuses on assessing the influence of two factors: the carbon nanotube amount and the sonication time. To determine the pure effects of these two factors in all pastes, the same amount of superplasticizers was applied, both used in sonication and in pastes. Three dosages of multi-walled carbon nanotubes (MWCNTs) equal to 0.05 wt%, 0.1 wt%, and 0.2 wt% and two sonication times of the solution of 20 min and 60 min were applied in the experimental programme. The results acquired with the carbon nanomaterials were compared with clean cement paste and cement paste with the addition of a polycarboxylate ether (PCE) superplasticizer.

This paper is divided into four sections. After this introduction, Section 2 describes the materials and methods used in the experimental program. The obtained results are presented and discussed in Section 3. Finally, conclusions from the experiments are listed in Section 4.

## 2. Materials and Methods

The cementitious composites used in the tests were prepared using Ordinary Portland cement CEM I 42.5 R (OPC), and multi-walled carbon nanotubes (MWCNTs) in a water suspension were made with a naphthalene-based superplasticizer, tap water, and a polycarboxylate-ether-based superplasticizer. The chemical and mineral composition of the tested cement are presented in Table 1 and Table 2, respectively. The basic physical and mechanical properties of OPC are depicted in Table 3.

The MWCNTs used in the study were NANOCYL NC7000 [31], which are tube-shaped materials, exclusively composed of carbon atoms, with an average length of 1.5 µm, an average diameter of 9.5 nm, and 90% purity. The basic properties of Nanocyl NC7000 multi-walled carbon nanotubes are listed in Table 4.

The suspension was prepared in distilled water with a naphthalene-based superplasticizer, which was found to be the best for ensuring a homogeneous suspension of carbon nanotubes [32]. In detail, a specified amount of MWCNTs (Table 5) was added to 100 g of distilled water and a constant amount of 4.5 g of naphthalene-based superplasticizer. The amount of superplasticizer for sonication was constant in all pastes, regardless of the amount of MWCNTs. Then, without using mechanical stirring, the mix was sonicated using an ultrasonic homogenizer Hielscher UP200St [33] with a constant 40% amplitude and a maximum frequency of 26 kHz. Two sonication times of 20 min or 60 min were applied. Aiming to reduce water evaporation, the total sonication time was divided into 30 s intervals, while the container was constantly cooled in an ice bath. The sonication parameters were derived based on the preliminary tests, while the amplitude was set as 40% because of the high heat generation at higher amplitudes, despite using the abovementioned cooling techniques.

In the experimental tests, the effects of two factors influencing the hydration process. the MWCNT dosage and the sonication time, were examined. As already mentioned, the sonication time was equal to 20 min or 60 min. Considering the dosage of multi-walled carbon nanotubes, the cementitious composites were prepared with 0.05 wt%, 0.1 wt% and 0.2 wt% of MWCNTs related to the cement weight. In all samples, the applied water/cement ratio was equal to 0.5. It should be pointed out that the water used to prepare the suspension was accounted for in the assumed w/c ratio. In all samples, a polycarboxylate-based superplasticizer in powdered form was directly added to the cement in an amount of 0.525% of the cement weight. It should be noted that the same amounts of superplasticizers in the pastes, regardless of the amount of MWCNTs, were used to investigate the pure effect of the nanotubes on the hydration process. Finally, the tested cement composites, along with the proper notations used in the discussion of the results, are listed in Table 5.

The heat generated during the hydration process as well as the rate of heat evolution was measured in an eight-channel isothermal calorimeter TAM Air, which is a high-precision device suitable for analysing the hydration reaction in cementitious composites [34]. The applied testing procedure was based on the European standard [35]. Hence, each sample of 5 g of the examined cementitious composite and distilled water in the amount compliant with Table 5 was placed in the TAM Air at least 24 h before the start of the measurement to achieve a temperature of the sample equal to the assumed curing temperature of 20 °C. The internal mixing method was adopted. The heat evolved was measured for 168 h. For each of the cementitious composites listed in Table 5, 3 samples were prepared, and the results in Section 3 show the average values of heat emission from these three measurements. In all cases, the determined standard deviation was lower than 5%.

## 3. Results and Discussion

The five periods of the hydration heat evolution are usually distinguished and described as the initial period, the induction period, the acceleration period, the retardation period, and the period of slow continued reaction. The initial period is characterised by rapid heat generation caused by the fast formation of an amorphous layer of hydration product around the cement grains. This initial hydration and dissolution are manifested as the first peak in the heat flow curve. Next, almost no reaction occurs in the induction period, and the heat flow drops rapidly to a very low level. Subsequently, in the acceleration period, the rate of reaction grows rapidly and reaches a main peak that corresponds to the hydration of tricalcium silicate (C_3_S) and the fast formation of C-S-H gel. In the deceleration period, hydration starts to slow down as less water is able to infiltrate into cement grains due to the growing C-S-H gel that traps water in its pores; therefore, a decrement in heat generation is observed. The last stage of the hydration process is a slow reaction of cement grains that are not yet fully covered in hydration products.

**Figure 1:**
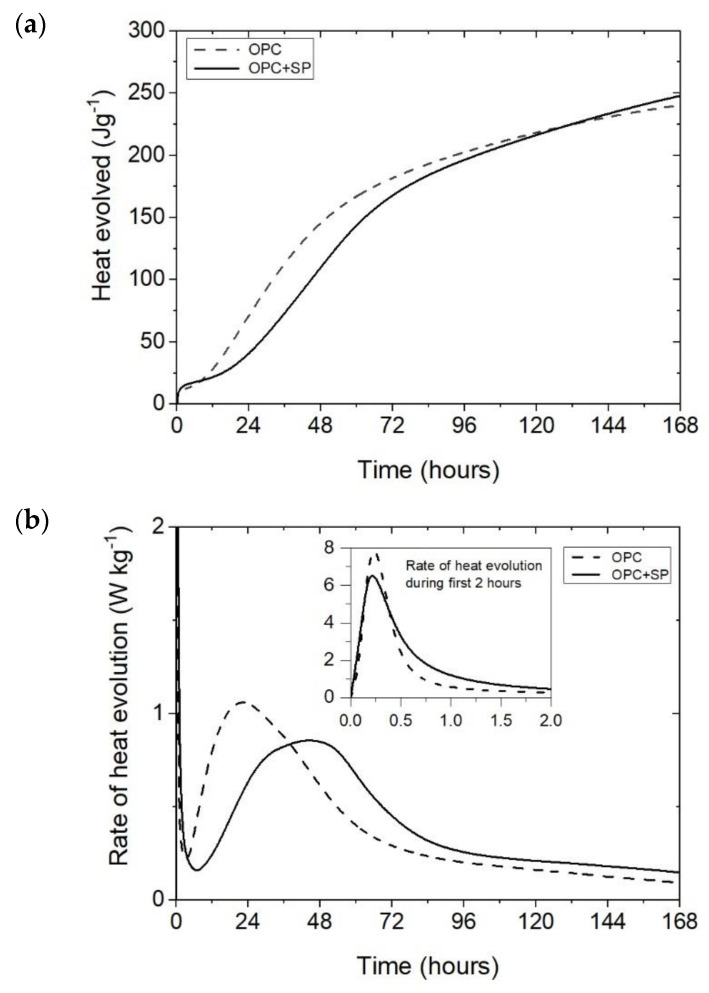
The heat evolved (**a**) and the rate of heat evolution (**b**) registered for OPC and OPC + SP.

**Figure 2:**
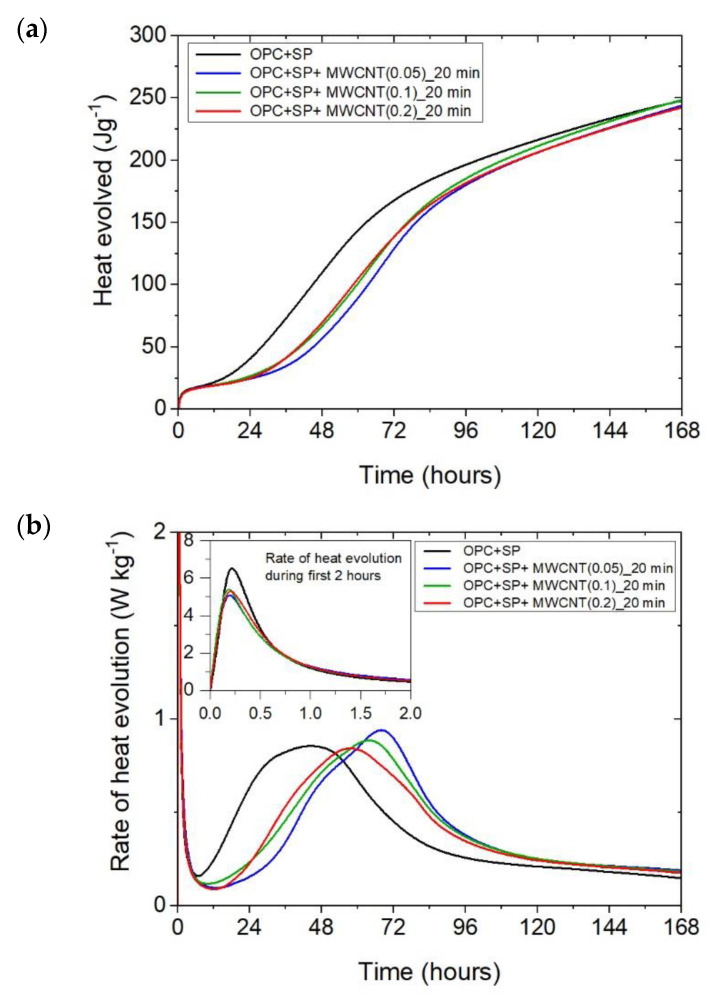
The heat evolved (**a**) and the rate of the heat of evolution (**b**) measured for OPC + SP with 0.05%, 0.1%, and 0.2% of MWCNTs and the sonication time equal to 20 min.

**Figure 3:**
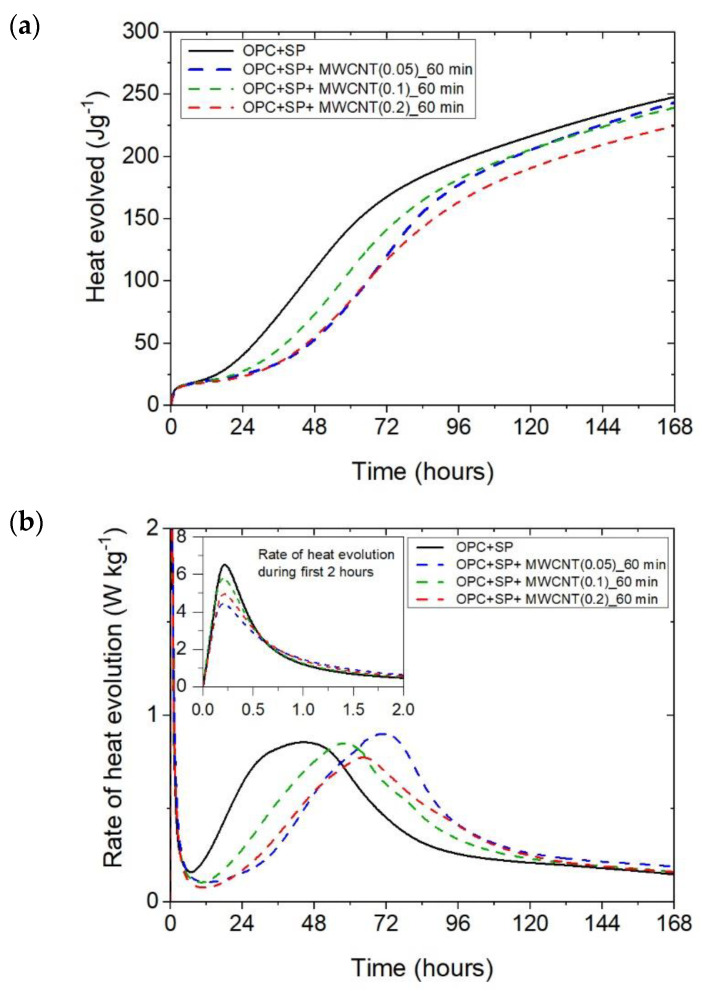
The heat evolved (**a**) and the rate of heat of evolution (**b**) measured for OPC + SP with 0.05%, 0.1%, and 0.2% of MWCNTs and the sonication time equal to 60 min.

**Figure 4:**
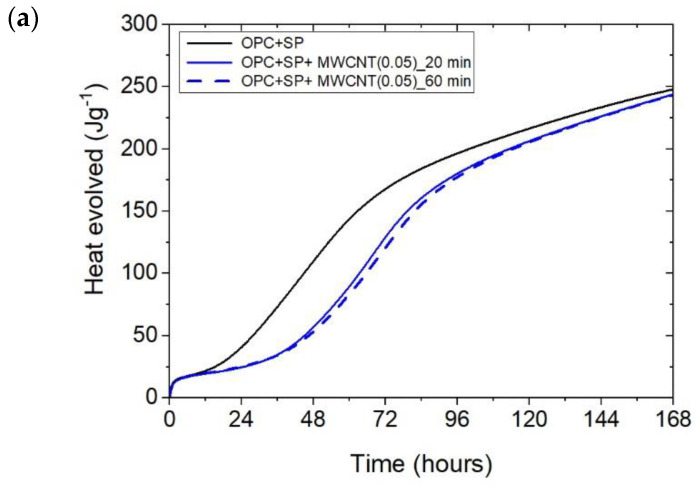

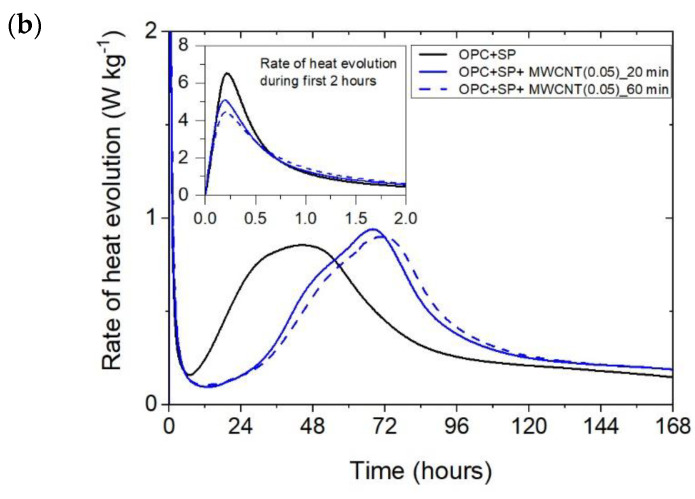
The heat evolved (**a**) and the rate of heat of evolution (**b**) registered for OPC + SP with 0.05 wt% of MWCNTs and two sonication times.

**Figure 5:**
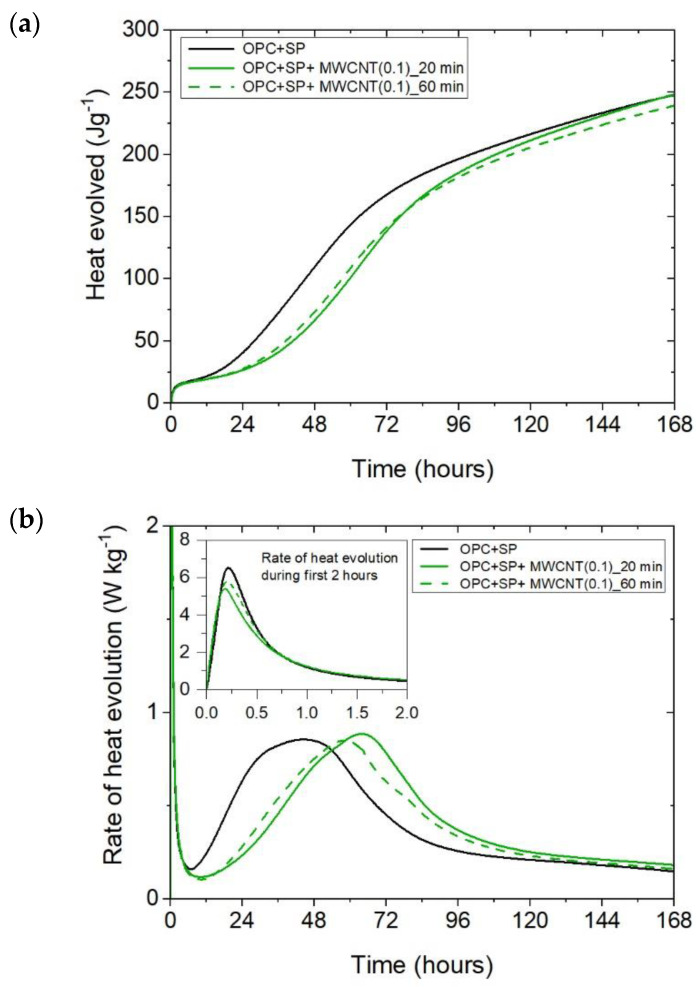
The heat evolved (**a**) and the rate of heat of evolution (**b**) registered for OPC + SP with 0.1 wt% of MWCNTs and two sonication times.

**Figure 6:**
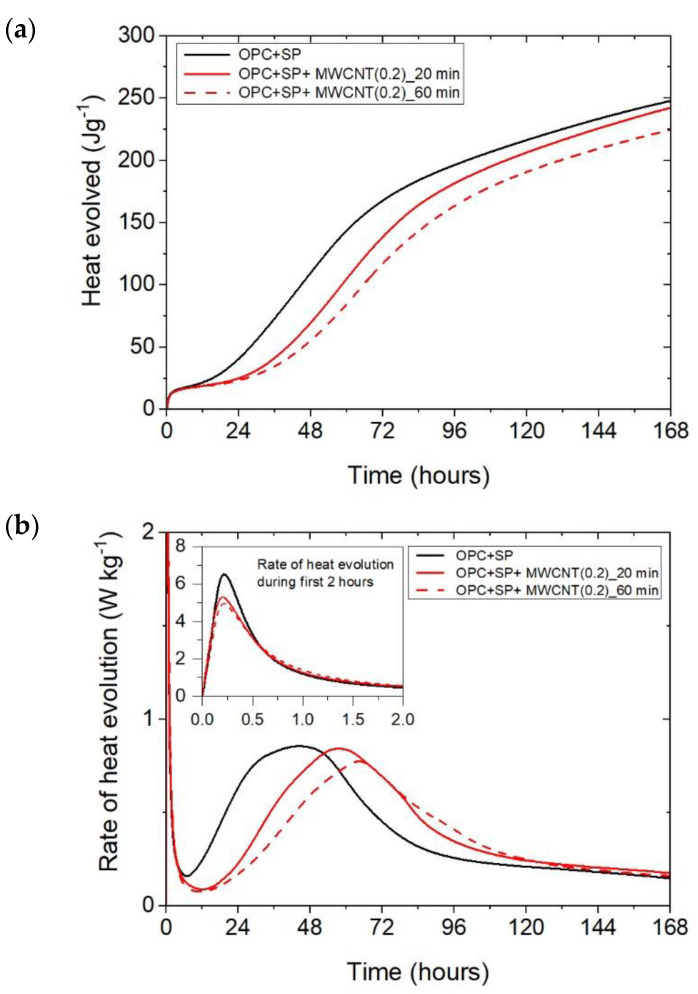
The heat evolved (**a**) and the rate of heat of evolution (**b**) registered for OPC + SP with 0.2 wt% of MWCNTs and two sonication times.

**Figure 7:**
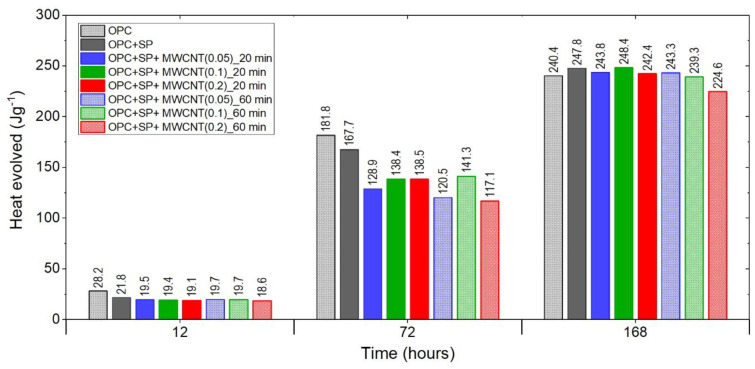
The heat evolved after 12, 72, and 168 h in the tested composites.

**Figure 8:**
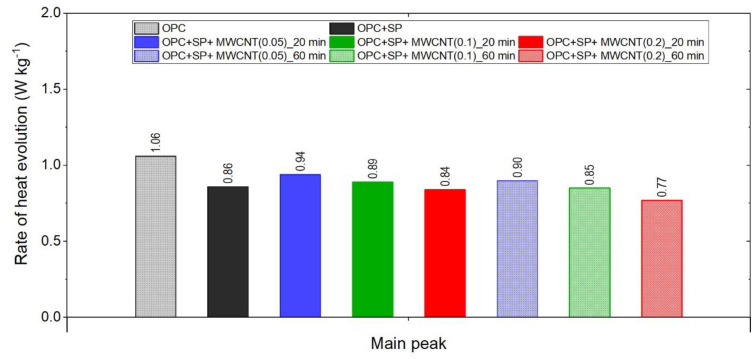
The main peak values of the heat evolution rate in the tested composites.

**Figure 9:**
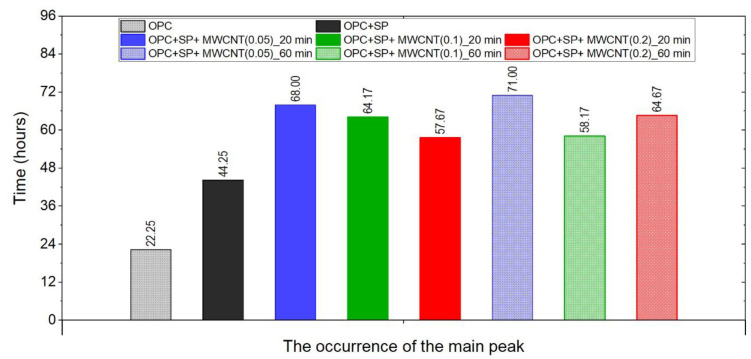
The times of the occurrence of the main peak of heat evolution rate in the tested composites.

The following section discusses the results of cumulative hydration heat and heat evolution rate measured in the tested composites in relation to the above-described phases of the hydration process. All results are presented in Figure 1, Figure 2, Figure 3, Figure 4, Figure 5, Figure 6, Figure 7, Figure 8 and Figure 9, while Table 6 provides characteristic values of heat evolved in the tested cementitious composites.

The effect of a polycarboxylate-ether-based superplasticizer, used mainly to ensure the appropriate consistency of composites with nanotubes, on the cumulative heat and the rate of heat evolution is presented in Figure 1. Not surprisingly, the addition of a superplasticizer (SP) affects the hydration of the cement paste. It can be seen in Figure 1a and Figure 7 and Table 6 that the 12 h heat emission of the composite with SP was lower by 22.70% compared with the sample without SP. During the progress of hydration, the discussed reduction in heat decreased, and for 72 h, it was only 7.76%. Finally, after 168 h, the amount of heat released was 3.08% higher for the sample with SP. In the initial period, the peak value of the composite with a superplasticizer (OPC + SP) was approximately 20% lower (Table 6) compared with the sample without a superplasticizer (OPC). Next, the addition of a superplasticizer distinctly retarded the acceleration period, and the main peak was noted 22 h later than in the composite without a superplasticizer. The main hydration peak was also significantly decreased in the presence of a superplasticizer. In detail, the main peak reached 0.86 W kg^−1^ in the presence of a superplasticizer compared with 1.06 W kg^−1^ in the sample without it (Figure 1b and Figure 8; Table 6).

It is important to highlight that the different nature of the hydration heat development described in the next paragraphs for the pastes with MWCNTs in comparison with the reference paste (OPC + SP) can be linked to the usage of a naphthalene-based superplasticizer during sonication, as the quantity of this superplasticizer remained consistent across all tested pastes with nanotubes. Therefore, the distinctions observed in the curves for different MWCNT concentrations and varying sonication durations in the results discussion were also attributed to the influences of these two factors.

The cumulative heat and the heat evolution curves obtained for cementitious composites with 0.05 wt%, 0.1 wt%, and 0.2 wt% of MWCNTs and two sonication times (20 min or 60 min) are depicted in Figure 2 and Figure 3. The characteristic values related to the released heat are listed in Table 6. To facilitate the interpretation of the results, the obtained values are also presented graphically in Figure 7, Figure 8 and Figure 9. All curves plotted in Figure 2 and Figure 3 show the distinct behaviours of the composites enhanced with MWCNTs compared with the sample without carbon nanotubes. Dissimilarity may be noted in all periods of the hydration heat evolution.

Considering the heat evolved in composites with 0.05 wt%, 0.1 wt%, and 0.2 wt% of MWCNTs and a sonication time of 20 min (Figure 2a), the results clearly show the lower heat emission in the early hours of the hydration process. All samples with MWCNTs and using a naphthalene-based superplasticizer exhibited lower values after 12 h with progressively higher differences of 10.55%, 11.01%, and 12.39% for MWCNT additions of 0.05 wt%, 0.1 wt%, and 0.2 wt%, respectively. However, after 72 h, the trend was reversed. The heat evolved was lower compared with the reference sample, but the biggest difference of 23.14% was now for the 0.05 wt% of MWCNTs, followed by 17.47% (0.1 wt% of MWCNTs) and 17.74% (0.2 wt% of MWCNTs), correspondingly less than the reference sample (OPC + SP). Finally, the heat emission after 168 h was at a similar level in all discussed composites (Table 6). MWCNTs and a naphthalene-based superplasticizer inclusion also affected the rate of heat evolution (Figure 2b). The local maximum in the initial period was 12.8–18.9% lower than in the reference sample, and the induction period of hydration was gradually prolonged with the decreased dosage of MWCNTs (Table 6). In detail, the time of the main peak was delayed in all composites with MWCNTs, with the biggest prolongation time being 23.75 h for 0.05 wt% of MWCNTs, 19.92 h for 0.1 wt%, and 13.42 h for 0.2 wt% (Table 6). Contrary to the first maximum, the main peak of the heat evolution rate of MWCNT composites did not show such a clear relationship compared with the reference sample. In the case of a composite with 0.5 wt% of MWCNTs, the maximum value was 34.9% higher than in the reference sample, while for the composite with 0.1 wt% of MWCNTs, the value was identical to the reference value (OPC + SP), and for the content of MWCNT equal to 0.2 wt%, it was lower by 4.65% (Figure 8).

The results for the three composites enhanced with MWCNTs and a sonication time of 60 min are depicted in Figure 3. The results are basically similar to those for the composites with a sonication time of 20 min, except for the composite with the highest MWCNT content of 0.2 wt%. Consequently, the heat that evolved after 12 h was lower in all composites with MWCNTs compared with the reference sample (Figure 3a and Figure 7; Table 6). The lowering of the heat released compared with the reference sample (OPC + SP) was the largest for the composite with 0.2 wt% of MWCNTs (14.68%), while for 0.05 wt% and 0.1 wt% contents of MWCNTs in the composite, the same reduction of 9.63% was noted. After 72 h, the heat evolved was lower by 28.15%, 15.74%, and 30.17% for the composites enhanced with 0.05 wt%, 0.1 wt%, and 0.2 wt% of MWCNTs, respectively. Interestingly, the composite with the highest MWCNT content, 0.2 wt%, evolved less heat after 168 h. This was approximately 9% less than the reference sample (Figure 7, Table 6). Analysing the initial hydration period, the extension of the sonication time from 20 min to 60 min did not significantly change the behaviour of the composites in the context of the heat release rate and the first maximum. The only observation was a greater impact of the amount of MCWNTs on the maximum value, but at the same time, there was no clear relation between the first peak and the amount of MCWNTs—for the maximum MCWNT content in the composite (0.2 wt%), an intermediate value of the first maximum was noted. Similarly, a non-progressive relationship was noted for the time of the main peak, which was delayed for all samples but occurred at 26.75 h, 13.92 h, and 20.42 h later for 0.05 wt%, 0.1 wt%, and 0.2 wt% compared with the reference sample, respectively. The same tendency was observed for the values of the main peak which, for the proper content of MWCNTs, were 4.65% higher for 0.05 wt%, 1.16% lower for 0.1 wt%, and 10.47% lower for 0.2 wt% in relation to the reference sample.

The effect of the sonication time for all composites with different amounts of MWCNTs is also compared in Figure 4, Figure 5 and Figure 6. It can be seen that sonication time affected the hydration process. Generally, the longer sonication time slightly reduced the amount of heat generated after 168 h. In detail, the reduction in the cumulative heat was 0.2%, 3.66%, and 7.34% in composites with 0.05 wt%, 0.1 wt%, and 0.2 wt% of MWCNTs, respectively (Table 6; Figure 7). The heat evolved after 12 h was at a very similar level, while after 72 h, the trend of a lower heat release with a longer sonication time was disturbed in the sample with 0.1 wt% of MWCNTs, where the heat emission was slightly higher by 2%. Considering the main hydration peak, in all samples, the longer sonication time decreased its value, and this effect was most significant in the composite with 0.2 wt% of MWCNTs, where the peak value was reduced from 0.84 W kg^−1^ to 0.77 W kg^−1^; therefore, by 8.33%. The effect of the sonication time on the occurrence of the main peak was without a clear trend. For composites with 0.05 wt% and 0.2 wt% of MWCNTs, the maximum occurred 3 h and 7 h later, respectively, with the longer sonication time. At the same time, the composite with 0.1 wt% of MWCNTs showed an earlier peak with the longer sonication time.

Finally, Figure 7 summarises the results for the heat evolved after 12 h, 72 h, and 168 h for all samples, including both reference samples. After 12 h, all composites with MWCNTs showed lower values of heat evolved than the reference samples. These lower values were at a similar level except for the sample with 0.2 wt% of MWCNTs and a 60 min sonication time, which had a significantly lower value.

After 72 h, the discrepancies between the MWCNT samples became more visible despite the same amount of a naphthalene-based superplasticizer used for sonication. There was a significant difference regarding the MWCNT dosages for both sonication times. For a sonication time of 20 min, the heat evolved grew with an increased amount of MWCNTs, with the difference between 0.1 wt% and 0.2 wt% being negligible. For a sonication time of 60 min, the sample with 0.1 wt% of MWCNTs exhibited a higher value of heat evolved than the others, while in the composite with 0.2 wt% of MWCNTs, the heat evolved was the lowest. It is important to note that the same composite with 0.2 wt% of MWCNTs after 20 min of sonication had the highest value of heat evolved, while after 60 min, the heat evolved was the lowest among the tested composites. Furthermore, at each MWCNT dosage, besides 0.1 wt%, the amount of heat evolved was higher after 20 min of sonication; however, it should be noted that the difference between the values of heat evolved for 0.1 wt% of MWCNTs was relatively small.

After 168 h, only one sample, denoted as OPC + SP + MWCNT(0.1)_20min, insignificantly exceeded the reference sample OPC + SP in values of heat evolved. Regarding the MWCNT dosage, after 60 min of sonication, the amount of heat evolved decreased with an increase in the MWCNT amount. After 20 min, the sample with 0.1 wt% of MWCNTs reached the highest value, slightly higher than OPC + SP, while 0.05 wt% of MWCNTs reached a substantially higher value than that with 0.2 wt% of MWCNTs. Considering the sonication time, for all dosage levels of MWCNTs, the samples with a shorter sonication time exhibited higher values of heat evolved.

The final comparison of main peak values is presented in Figure 8. All composites exhibited lower values of heat evolved at the main peak compared with the reference samples without a naphthalene-based superplasticizer used for sonication. The relationship with the SP reference sample is not unambiguous; three composites had a higher peak value and three had lower ones. Lower values were generally achieved by composites with a higher MWCNT content (0.2 wt% with both sonication times and 0.1 wt% at 60 min of sonication). For all samples with MWCNTs, there was a clear influence of the sonication time on the main peak value, with a time of 60 min giving lower values. Regarding the MWCNT dosage, the peak value decreased with the higher dosage of MWCNTs. At the same time, the decreases in the maximum value were not large as it was approximately 5.6% with a subsequent increase in the MWCNT content by 0.05% (from the nanotube content of 0.05 to 0.1%) or by 0.1% (from the nanotube content of 0.1 to 0.2%).

The time occurrence of the main peak for all the samples is depicted in Figure 9. All of the samples with MWCNTs exhibited retardation in the occurrence of the main peak, and the composites with the lowest amount of MWCNTs (0.05 wt%) exhibited the most delay. The sonication time influenced the peak time. For the samples with 0.05 wt% and 0.2 wt% of MWCNTs, the time increased with longer sonication, while for the samples with 0.1 wt% of MWCNTs, it decreased. Regarding the dosage, for a sonication time of 20 min, the main peak occurred sooner with an increment in MWCNTs. while for 60 min of sonication, this trend was valid only for 0.05 wt% and 0.2 wt% of MWCNTs. The sample with 0.1 wt% of MWCNTs exhibited different behaviour.

Finally, it can be noted that the influence of carbon nanotubes (MWCNTs) on the hydration process in cementitious materials involves several mechanisms, primarily attributed to the unique structural and surface properties of MWCNTs. Hence, the influence of MWCNTs on the hydration process is multifaceted, involving interactions at the molecular level, changes in water dynamics, and modifications to the cement matrix structure. Considering the obtained results as well as the findings from the related works [7,17,26,27,28,29], the following mechanisms contributing to the hydration process may be mentioned:

- Although MWCNTs are not chemically active during the hydration process, they can act as bridges between cement particles, and this bridging effect contributes to the course of the hydration process.
- MWCNTs may hinder water diffusion within the cement matrix due to their hydrophobic nature. This can lead to a more controlled and gradual release of water during the hydration process, influencing the kinetics of cement hydration.
- The negative effect of MWCNTs on the induction phase may be linked to the steric effect of the nanotubes in the paste, adsorbing on the cement particles, and thus hindering early hydration by restricting water access to cement grains. The place for adsorption is limited, and thus, the delay in hydration is almost similar for all samples with MWCNTs, and for higher amounts of MWCNTs, it is additionally offset by the higher amounts of MWCNTs providing more surfaces for hydration products and thus a shorter induction phase.
- Generally, superplasticizers delay hydration; hence, a naphthalene-based superplasticizer used during sonication also delays it. Nevertheless, the presence of MWCNTs combined with superplasticizers can create steric hindrance effects. This hindrance can additionally delay the hydration of MWCNT-enhanced cement paste by affecting the mobility of water molecules and impeding their access to reactive sites.
- The different effects of the sonication time can be connected to the fact that while short sonification can disperse the nanotubes and thus prevent their agglomeration, if the sonication is prolonged, there is a risk of breaking the long nanotubes into smaller pieces and, more importantly, their unravelling. This increases the surface area of the nanotubes and may provide more steric disruption for the hydration products and increased water absorption by the MWCNTs, thus impeding hydration. It should be noted, however, that this effect is dependent on the amount of MWCNTs in the mixture.

It can be inferred that these mechanisms induced varied heat release patterns in the tested composites compared with the reference composite without MWCNTs, leading to delayed hydration but an increase in the strength of the hydration peak value. Simultaneously, it is evident that these effects were contingent upon the quantity of multi-walled carbon nanotubes (MWCNTs). Specifically, as the amount of MWCNTs exceeded a certain threshold, defined as optimal [27], the main hydration peak decreased with a simultaneous smaller hydration delay due to the agglomeration of carbon nanotubes within the cement matrix. This was especially visible when comparing the composite with MWCNT addition of 0.05 wt% with the two composites enhanced with MWCNTs in amounts of 0.1 and 0.2 wt% (Figure 2b). At the same time, an extended sonication time may also improve the distribution of MWCNTs and prevent the agglomeration of MWCNTs, but in the presented results, this effect was slightly visible only for the composite with 0.1 wt% of MWCNTs (Figure 5b). Hence, another effect related to the breaking of the long nanotubes into smaller pieces was noticed in these tests, especially for the high content of MWCNTs (Figure 6b).

## 4. Conclusions

The presented results indicate that multi-walled carbon nanotubes (MWCNTs) influence the hydration process in cementitious composites, modifying the heat release curves and the amount and timing of hydrated compound formation. Although the naphthalene-based superplasticizer used to disperse MWCNTs also influenced the hydration of cement, its amount was constant among all the samples, and the results varied between different dosages and sonication times of the suspensions. Hence, if only the superplasticizer used in the suspension causes differences in the hydration kinetics, no variation in the results should be visible between the different amounts of MWCNTs as well as sonication times.

The main conclusions related to the effect of MWCNTs on the hydration process can be drawn as follows:

- In terms of heat emission, the addition of multi-walled carbon nanotubes and the naphthalene-based superplasticizer used to disperse MWCNTs generally reduced its value at the early stage of hydration. The influence was most significant after 72 h, which corresponded to the acceleration period associated with the rapidly growing rate of reaction and reaching a main peak for samples with MWCNTs and the naphthalene-based superplasticizer addition.
- The MWCNTs and the naphthalene-based superplasticizer used to disperse MWCNTs reduced the first peak value in the initial stage of hydration and significantly delayed (even up to almost 27 h) the acceleration period compared with the reference sample without this superplasticizer. Simultaneously, the main hydration peaks of the composites with the 0.05 wt% MWCNT dosage reached higher values compared with the mentioned reference sample. With the highest content of MWCNTs, i.e., 0.2 wt%, the main peak value of the heat evolution rate was lower than that of the mentioned reference sample. In the case of using 0.1 wt% of MWCNTs in the composite, the peak value related to the reference sample depended on the sonication time, and so, at 20 min it was higher, and at 60 min it was slightly lower. Hence, the relationship is not clear.
- The amount of MWCNTs and the sonication time also affect the hydration heat emission and the rate of reaction. Despite the same amount of the naphthalene-based superplasticizer used for sonication, the rate of the hydration heat at the main peak decreased with the increase in the MWCNT dosage in all pastes. Simultaneously, a longer sonication time resulted in lower values of heat evolved after 168 h, lower values of the main peak of heat rate evolution for all samples, and generally longer retardation of the main peak occurrence. When comparing the relative impacts of the MWCNT dosages and sonication time on the hydration process, the results suggest that the primary factor influencing the outcome is the MWCNT content. An extended sonication time only amplifies these effects, as indicated by the presented results.

Finally, it should be mentioned that the hydration process in composites with MWCNTs is not fully recognised and understood. Furthermore, the research results are sometimes discrepant and indicate divergent effects of carbon nanotubes on the hydration process, from no effect, accelerating the hydration process, to retarding it. While there is a consensus that carbon nanotubes primarily function as solid nanofillers without undergoing chemical interactions with cement components and have limited influence on hydration from this perspective, this study validates certain observations in the existing literature regarding the potential impact of nanotubes on hydration. This influence is likely attributable to physical phenomena. Therefore, the presented results may be a contribution to this discussion and understanding of the hydration of composites with carbon nanotubes.

## Figures and Tables

**Table 1:** Chemical composition of Ordinary Portland cement CEM I 42.5R (OPC).

Component	Content (%)
Loss on ignition	2.66
Insoluble residue	0.73
SiO_2_	20.16
Al_2_O_3_	5.30
Fe_2_O_3_	2.69
CaO	63.37
MgO	1.41
SO_3_	2.63
Na_2_O	0.17
K_2_O	0.81
Cl	0.095

**Table 2:** Mineral composition of Ordinary Portland cement CEM I 42.5R (OPC).

Component	Content (%)
Portland clinker:	95.7
-C_3_S	68.5
-C_2_S	11.8
-C_3_A	10.5
-C_4_AF	8.3
-Free Cao	0.95
Non-clinker components (limestone and gypsum)	4.3

**Table 3:** Physical properties and strength of Ordinary Portland cement CEM I 42.5R (OPC).

Property	Value
Compressive strength (MPa)	
-After 2 days	28.9
-After 28 days	57.8
Water demand (%)	27.6
Specific surface (cm^2^/g)	3510
Initial setting time (min)	175
Soundness (mm)	0.4

**Table 4:** Properties of Nanocyl NC7000 [31].

Property	Value
Average diameter (nm)	9.5
Average length (μm)	1.5
Carbon purity (%)	90
Transition metal oxide (%)	<1
Specific area (m^2^/g)	250–300
Volume resistivity (Ω cm)	10^−4^

**Table 5:** The tested cementitious composites.

Cementitious Composite	w/c	Superplasticizer (wt%)	MWCNT * (wt%)	Sonication Time (min)
OPC	0.5	0.525	-	-
OPC + SP			-	-
OPC + SP + MWCNT(0.05)_20 min			0.05	20
OPC + SP + MWCNT(0.1)_20 min			0.1	20
OPC + SP + MWCNT(0.2)_20 min			0.2	20
OPC + SP + MWCNT(0.05)_60 min			0.05	60
OPC + SP + MWCNT(0.1)_60 min			0.1	60
OPC + SP + MWCNT(0.2)_60 min			0.2	60

* The amount of superplasticizer for sonication (4.5 g of naphthalene-based superplasticizer) was the same in all pastes, regardless of the amount of MWCNTs.

**Table 6:** Characteristic values of the heat evolved in the tested cementitious composites.

Cementitious Composite	The Heat Evolved (J g^−1^)				The Peak Value of Heat Evolution Rate/Time of the Occurrence, (W kg^−1^/h)	
	12 h	41 h	72 h	168 h	First Peak	Main Peak
OPC	28.2	127.9	181.8	240.4	8.32/0.25	1.06/22.25
OPC + SP	21.8	88.3	167.7	247.8	6.62/0.17	0.86/44.25
OPC + SP + MWCNT(0.05)_20 min	19.5	42.1	128.9	243.8	5.37/0.17	0.94/68.00
OPC + SP + MWCNT(0.1)_20 min	19.4	50.6	138.4	248.4	5.77/0.17	0.88/64.17
OPC + SP + MWCNT(0.2)_20 min	19.1	52.0	138.5	242.4	5.54/0.17	0.84/57.67
OPC + SP + MWCNT(0.05)_60 min	19.7	40.7	120.5	243.3	4.62/0.17	0.90/71.00
OPC + SP + MWCNT(0.1)_60 min	19.7	56.0	141.3	239.3	6.02/0.17	0.85/58.17
OPC + SP + MWCNT(0.2)_60 min	18.6	42.1	117.1	224.6	5.10/0.17	0.77/64.67

## Data Availability

Data are contained within the article.
